# MicroRNA‐134‐5p inhibition rescues long‐term plasticity and synaptic tagging/capture in an Aβ(1–42)‐induced model of Alzheimer’s disease

**DOI:** 10.1111/acel.13046

**Published:** 2019-10-17

**Authors:** Nimmi Baby, Nithyakalyani Alagappan, Shaikali Thameem Dheen, Sreedharan Sajikumar

**Affiliations:** ^1^ Department of Physiology Yong Loo Lin School of Medicine National University Health System National University of Singapore Singapore; ^2^ Centre for Life Sciences Life Sciences Institute, Neurobiology Programme National University of Singapore Singapore; ^3^ Department of Anatomy Yong Loo Lin School of Medicine National University Health System National University of Singapore Singapore

**Keywords:** Alzheimer's disease, Aβ(1–42), brain‐derived neurotrophic factor, cAMP response element‐binding protein, long‐term potentiation, miRNA, synaptic tagging

## Abstract

Progressive memory loss is one of the most common characteristics of Alzheimer's disease (AD), which has been shown to be caused by several factors including accumulation of amyloid β peptide (Aβ) plaques and neurofibrillary tangles. Synaptic plasticity and associative plasticity, the cellular basis of memory, are impaired in AD. Recent studies suggest a functional relevance of microRNAs (miRNAs) in regulating plasticity changes in AD, as their differential expressions were reported in many AD brain regions. However, the specific role of these miRNAs in AD has not been elucidated. We have reported earlier that late long‐term potentiation (late LTP) and its associative mechanisms such as synaptic tagging and capture (STC) were impaired in Aβ (1–42)‐induced AD condition. This study demonstrates that expression of miR‐134‐5p, a brain‐specific miRNA is upregulated in Aβ (1–42)‐treated AD hippocampus. Interestingly, the loss of function of miR‐134‐5p restored late LTP and STC in AD. In AD brains, inhibition of miR‐134‐5p elevated the expression of plasticity‐related proteins (PRPs), cAMP‐response‐element binding protein (CREB‐1) and brain‐derived neurotrophic factor (BDNF), which are otherwise downregulated in AD condition. The results provide the first evidence that the miR‐134‐mediated post‐transcriptional regulation of CREB‐1 and BDNF is an important molecular mechanism underlying the plasticity deficit in AD; thus demonstrating the critical role of miR‐134‐5p as a potential therapeutic target for restoring plasticity in AD condition.

## Graphical abstract

Late long‐term potentiation (late LTP) and synaptic tagging and capture (STC) are impaired in Aβ (1–42)‐treated AD hippocampus. miR‐134‐5p expression is significantly upregulated in AD hippocampus which induces impairment in late LTP and STC by post‐transcriptionally regulating the expression of plasticity‐related genes CREB‐1 and BDNF. Inhibition of miR‐134‐5p using miR‐134‐5p specific antagomir (miR‐134i) upregulates the expression of CREB‐1 and BDNF which is otherwise downregulated in AD thereby, reinstating late LTP and STC in AD hippocampus.

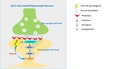

## 1 INTRODUCTION

Alzheimer's disease (AD), one of the major neurodegenerative disorders characterized by progressive memory loss and cognitive impairment, is mainly caused by the accumulation of amyloid β peptide (Aβ) and neurofibrillary tangles (Chen et al., 2017; Hardy & Selkoe, 2002). Aβ type (1–42) has been identified as a primary cause for amyloid plaque formation, loss of neurons and synaptic failure in the hippocampus, leading to deficits in synaptic plasticity and memory (Chen et al., 2000; Selkoe & Hardy, 2016; Sharma, Dierkes, & Sajikumar, 2017; Sheng, Sabatini, & Südhof, 2012). Exogenous application of Aβ (1–42) impairs long‐term potentiation (LTP), a cellular correlate of memory and synaptic tagging/capture (STC), a model to study associative plasticity in the hippocampus (Jiang et al., 2015; Krishna, Behnisch, & Sajikumar, 2016; Lei et al., 2016; Ma et al., 2014; Sharma et al., 2017). The STC model states that memory formation is an associative and time‐dependent process (Frey & Morris, 1997; Redondo & Morris, 2011). In the STC model, a “tag” set by a weak stimulus or a weak memory trace “captures” the plasticity‐related proteins (PRPs) produced by a strong stimulus or a strong memory trace in two independent synaptic inputs of the same neuronal population. The interaction between the tag and PRPs results in the consolidation of memory (Redondo & Morris, 2011).

cAMP response element‐binding protein (CREB), a transcription factor, and brain‐derived neurotrophic factor (BDNF), known to be a plasticity protein, are two important PRPs that work as mediators of functional and structural plasticity (Caracciolo et al., 2018; Korte et al., 1995; Sajikumar & Korte, 2011). CREB is known to have a pivotal role in neuronal excitability (Caracciolo et al., 2018; Yu, Oh, & Disterhoft, 2017) and functions as a positive regulator of LTP and memory formation (Kida, 2012). BDNF maintains late LTP, late LTD and STC (Korte et al., 1995; Sajikumar & Korte, 2011). BDNF has been shown to have a neuroprotective effect against the toxicity induced by Aβ peptides (Arancibia et al., 2008; Caccamo, Maldonado, Bokov, Majumder, & Oddo, 2010). Further, downregulation of CREB and BDNF expression is associated with AD conditions where plasticity is impaired (Pugazhenthi, Wang, Pham, Sze, & Eckman, 2011; Sharma et al., 2017). However, the key molecular mechanism regulating the expression of these PRPs in AD condition remains unclear and needs to be elucidated.

Recently, microRNAs (miRNAs) have emerged as important epigenetic regulators of synaptic plasticity (Costa‐Mattioli, Sossin, Klann, & Sonenberg, 2009; Korte & Schmitz, 2016; Smalheiser & Lugli, 2009). miRNAs, approximately 22 nucleotides long, are endogenous, noncoding RNAs that bind to the 3’untranslated region (3’UTR) of its target messenger RNAs (mRNAs) and suppress its expression either by promoting mRNA degradation or by preventing translation (Lim et al., 2005). miRNAs modulate synaptic plasticity by binding to dendritic mRNAs, thus modulating local protein synthesis at synapses in an activity‐dependent manner (Korte & Schmitz, 2016; Schratt et al., 2006). Newly synthesized miRNAs are transported to synapses where they downregulate the expression of target proteins including PRPs. Hence, inhibiting the expression of these miRNAs leads to an increase in newly synthesized proteins that consequently impose structural and functional changes to the synapses, resulting in the maintenance of late LTP for hours (Korte & Schmitz, 2016).

Interestingly, differential expression of miRNAs is reported in various AD brain regions (Cogswell et al., 2008; Moradifard, Hoseinbeyki, Ganji, & Minuchehr, 2018; Nunez‐Iglesias, Liu, Morgan, Finch, & Zhou, 2010). A recent miRNA profiling study revealed that miRNA‐134 (miR‐134) expression was upregulated in AD patient samples (Moradifard et al., 2018). Overexpression of miR‐134 in hippocampal neurons led to a reduction in dendritic spine size (Schratt et al., 2006) and miR‐134 has also been shown to be involved in dendritogenesis in vivo*,* facilitating its role in synapse development and plasticity (Christensen, Larsen, Kauppinen, & Schratt, 2010). Further, miR‐134 was shown to mediate LTP and synaptic plasticity through the Sirtuin1‐CREB‐BDNF pathway in the hippocampus (Gao et al., 2010). Since it is not clear if the upregulation of miR‐134 expression in AD patients (Moradifard et al., 2018) causes plasticity deficit, in the present study, we have investigated the functional role of miR‐134‐5p in regulating long‐term plasticity and cellular associativity in Aβ (1–42)‐treated hippocampal CA1 pyramidal neurons.

## 2 MATERIAL AND METHODS

### 2.1 Electrophysiology

A total of 180 transverse acute hippocampal slices (400 µm thick) from 100 adult male Wistar rats (5–7 weeks old) and 30 transverse acute hippocampal slices (400 µm thick) from three aged male mice (C57BL/6J, 16–18 months old) were used for electrophysiological experiments. We avoided using female rats and mice for our experiments primarily because hormonal alterations during the oestrous cycle can affect synaptic plasticity measurements (Monfort, Gomez‐Gimenez, Llansola, & Felipo, 2015; Qi et al., 2016; Warren, Humphreys, Juraska, & Greenough, 1995). Animals were housed under 12h light/12h dark conditions with food and water available ad libitum. All experimental procedures using animals were performed in accordance with the protocols approved by the Institutional Animal Care and Use Committee (IACUC) of the National University of Singapore (protocol number: R16‐0135). Briefly, the animals were decapitated after anesthetization using CO_2_. The brains were quickly removed and cooled in 4°C artificial cerebrospinal fluid (ACSF) that contained the following (in millimolar): 124 NaCl, 3.7 KCl, 1.0 MgSO4 .7H2O, 2.5 CaCl2, 1.2 KH2PO4, 24.6 NaHCO3 and 10 D‐glucose, equilibrated with 95% O_2_–5% CO_2_ (carbogen; total consumption 16 L/hr), and acute hippocampal slices were prepared from the right hippocampus using a manual tissue chopper. Hippocampal slices were then transferred onto the interface brain slice chamber (Scientific Systems Design) and incubated for three hours at 32°C with ACSF before the electrophysiology studies. Slices were treated with 200 nM Aβ (1–42) oligomers (Anaspec Inc) in a similar manner described in our previous reports (Krishna et al., 2016; Sharma et al., 2017) and 1 µM miR‐134 inhibitor (miR‐134i) oligonucleotide (AUM‐ANT‐A‐500 FANA miR‐134‐5p‐1 Inhibitor, AUM Biotech, LLC) or 1 µM scrambled miR‐134 inhibitor (FANA scrambled miR‐134 Inhibitor, AUM Biotech, LLC) at a flow rate of 1 ml/min of ACSF and 16 L/hr of carbogen for three hours during the incubation time. The entire process of animal dissection, hippocampal slice preparation and placement of slices on the chamber was done within approximately five minutes to ensure that hippocampal slices were in good condition for electrophysiology studies (Shetty et al., 2015). Since the number of aged mice were limited, both right and left hippocampus and a total of five interface chambers were used simultaneously to conduct five different experiments from each mouse.

**Figure 1:**
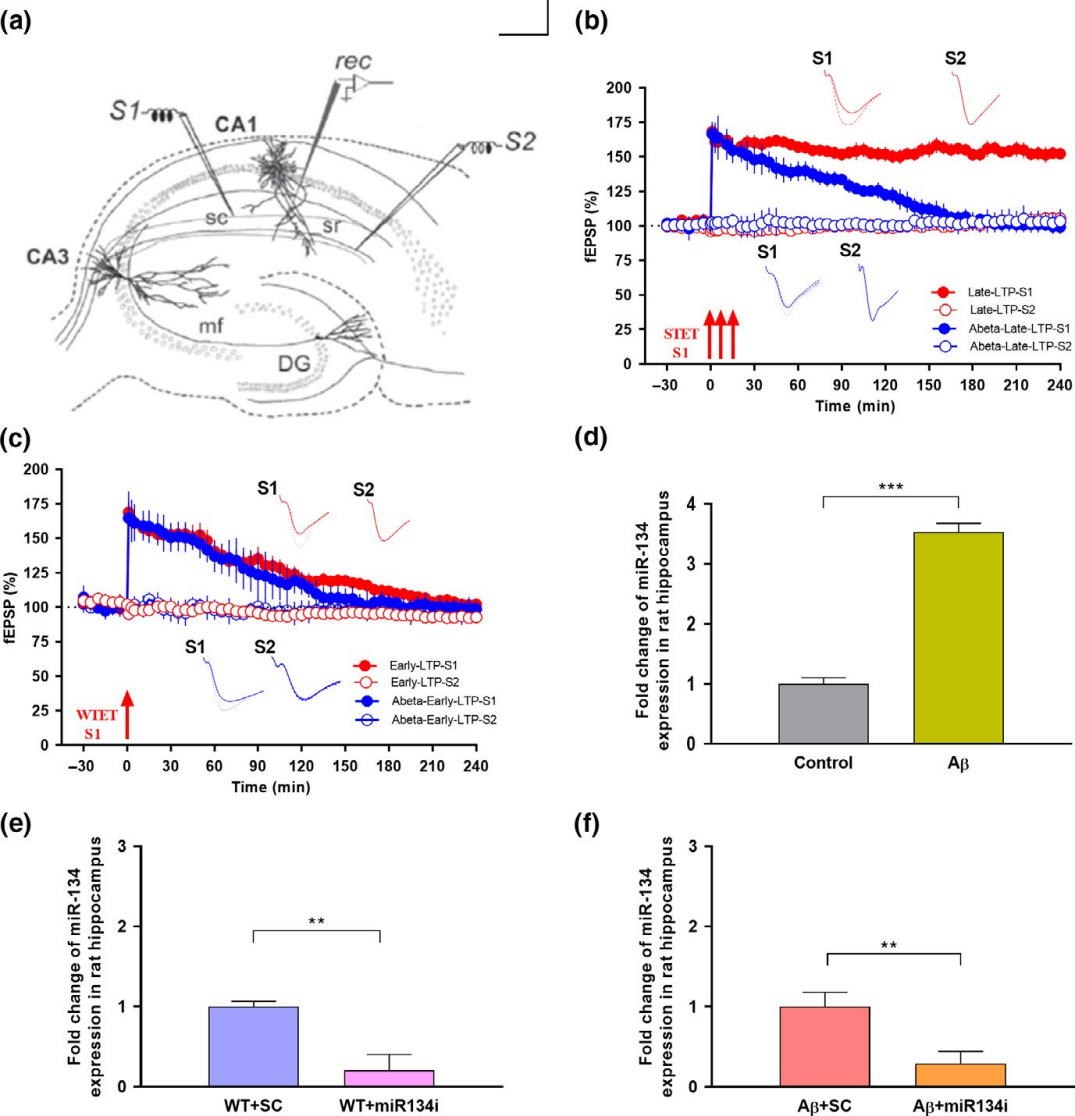
miR‐134‐5p expression in Aβ (1–42)‐treated rat hippocampus: (a) Schematic representation of the positioning of electrodes in the CA1 region of a transverse hippocampal slice. Recording electrode (rec) positioned in CA1 apical dendrites was flanked by two stimulating electrodes S1 and S2 in stratum radiatum (sr) to stimulate two independent Schaffer collateral (sc) synaptic inputs of the same neuronal population. (b) Late long‐term potentiation (late LTP) was maintained for 4 hr when a strong tetanization (STET) was applied to S1 (red closed circles). However, basal potential in S2 (red open circles) remained stable in wild‐type control slices (*n* = 7). STET application in S1 (blue closed circles) in Aβ (1–42) (200 nM) pretreated slices displayed impaired late LTP. Control potentials from S2 (blue open circles) remained stable throughout the recording (*n* = 7). (c) Induction of early LTP in S1 (red closed circles and blue closed circles) using a weak tetanization (WTET) protocol in both wild‐type control and Aβ (1–42) (200 nM) pretreated slices resulted in early LTP (red closed circles and blue closed circles, *n* = 7). Control potentials from S2 (red open circles and blue open circles) remained stable throughout the recording. All data presented as mean ± *SEM*. (d) qRT‐PCR analysis showed that miR‐134 expression was significantly increased in Aβ‐treated rat hippocampus by 3.5‐fold in comparison to wild‐type control rat hippocampus. Each sample was measured in duplicates and the expression of miR‐134‐5p was normalized to the expression levels of a miRNA reference gene, miR‐103‐3p and presented as mean ± *SD*. Significant difference between the group control versus Aβ is indicated by ***p ˂ .001, (student's *t* test, 12 slices each from 3 different biological samples, *n* = 3). (e‐f) Knockdown efficiency of miR‐134‐5p inhibitor in wild‐type and Aβ (1–42)‐treated rat hippocampal slices: (e) qRT‐PCR analysis showing a significant decrease in miR‐134‐5p expression in wild‐type slices treated with miR‐134i when compared to miR‐134‐5p scrambled inhibitor (SCi) treated wild‐type slices. (f) qRT‐PCR analysis showing a significant reduction of miR‐134‐5p expression in miR‐134i + Aβ (1–42) co‐treated rat hippocampal slices compared to SCi + Aβ(1–42) co‐treated slices. The data were normalized with miR‐103a‐3p, an internal control and presented as mean ± *SD*. Significant differences between the groups: WT + SCi versus WT + miR‐134i and SCi + Aβ versus miR‐134i + Aβ are indicated by *p ˂ .01 (student's *t* test, 12 slices each from 3 different biological samples, *n* = 3). The three arrows represent strong tetanization (STET) applied for inducing late LTP. Single arrow represents weak tetanization (WTET) applied for inducing early LTP. Insets in each graph represent typical fEPSP traces recorded 15 min before (continuous line), 30 min after (dotted line) and 240 min after (broken line) the induction of LTP. Calibration bar for all analog sweeps: vertical: 3 mV; horizontal: 5 ms

In all the electrophysiological recordings, two‐pathway experiments were performed. Two monopolar lacquer‐coated stainless steel electrodes (5MΩ; AM Systems, Sequim) were positioned at an adequate distance within the stratum radiatum of the CA1 region for stimulating two independent synaptic inputs S1 and S2 of one neuronal population, thus evoking field excitatory postsynaptic potentials (fEPSP) from Schaffer collateral/commissural‐CA1 synapses (Figure 1a). Pathway specificity was tested using the method described in (Sajikumar & Korte, 2011). One electrode (5MΩ; AM Systems) was placed in the CA1 apical dendritic layer for recording fEPSP. The signals were amplified by a differential amplifier (Model 1,700; AM Systems), digitized using a CED 1,401 analog‐to‐digital converter (Cambridge Electronic Design), and monitored online. After the pre‐incubation period, a synaptic input–output curve (afferent stimulation vs. fEPSP slope) was generated. Test stimulation intensity was adjusted to elicit fEPSP slope of 40% of the maximal slope response for both synaptic inputs S1 and S2. To induce late LTP, a “strong” tetanization (STET) protocol consisting of three high frequency stimulations of 100 pulses at 100 Hz (single burst, stimulus duration of 0.2 ms per polarity), with an inter‐train interval of 10 min, was used. To induce early LTP, a “weak” tetanization (WTET) protocol consisting of a single stimulus train of 21 pulses at 100 Hz (stimulus duration of 0.2 ms per polarity) was used (Shetty et al., 2015). In all experiments, a stable baseline was recorded for at least 30 min using four 0.2‐Hz biphasic constant‐current pulses (0.1 ms per polarity) at each time point.

### 2.2 Pharmacology

In vitro oligomer preparation of Aβ (1–42) peptide (AnaSpec, Fremont) was carried out 24 hr before the start of the experiment as reported previously (Krishna et al., 2016; Sharma et al., 2017; Stine, Dahlgren, Krafft, & LaDu, 2003). Briefly, Aβ (1–42) peptide films prepared in hexafluoroisopropanol (HFIP) were stored at −20°C. The peptide films were dissolved in dimethyl sulfoxide (DMSO) followed by DMEM/F‐12 without phenol red and were then stored at 4°C for 24h to allow the oligomerization of the peptide. The final concentration used for Aβ (1–42) was 200 nM (Krishna et al., 2016; Sharma et al., 2017; Stine et al., 2003). The miR‐134 antagomir (AUM‐ANT‐A‐500, FANA mir‐134‐5p‐1 Inhibitor, AUM Biotech, LLC) used in this set of experiments is a chemically modified, single‐stranded oligonucleotide sequence complementary to miR‐134‐5p. The target sequence used for miR‐134‐5p is UGUGACUGGUUGACCAGAGGGG. The transfection of miR‐134‐5p inhibitor in hippocampal slices was performed according to the manufacturer's instruction. We have used four miR‐134‐5p inhibitor constructs, out of which miR‐134‐5p‐1 construct showed maximum knockdown efficiency (80%) at the concentration of 1 µM. The inhibitor constructs used were specific to tissue slices and we observed a rapid effect in synaptic plasticity, probably due to direct incorporation of miR‐134 antagomirs into the acute hippocampal neurons. The stock solution was prepared in deionized water to a concentration of 20 µM. Working solutions of varying concentrations (5 µM, 2.5 µM and 1 µM) of miR‐134 inhibitor were prepared by diluting different volumes of the stock solution in ACSF. miR‐134 inhibitor (of all stated concentrations) was bath applied to hippocampal slices for 3 hr; subsequently, baseline potentials were recorded for 30 min followed by the application of the tetanization protocol. The control baseline potentials were not stable with the bath‐application of 5 µM and 2.5 µM miR‐134 inhibitor throughout the electrophysiology recordings. Hence, a final concentration of 1 µM miR‐134 inhibitor was used for this study. On the other hand, the scrambled inhibitor is a random sequence of oligonucleotides not complementary to miR‐134‐5p (AUM‐S500, AUM Biotech, LLC). The protein synthesis inhibitors, emetine dihydrochloride hydrate (Sigma‐Aldrich) and anisomycin (Tocris Biosciences, Bristol, UK), were stored as concentrated stock solutions of 20 μM in water and 25 μM in DMSO, respectively (Sajikumar, Navakkode, Korz, & Frey, 2007). NMDA receptor antagonist AP5 (Tocris Biosciences, Bristol, UK) was stored as 50 μM stock solution in water. For more details about drug application, see experimental design depicted in Figure S1 A‐C.

### 2.3 RNA isolation

Quantitative analysis of miR‐134‐5p, CREB‐1 and BDNF expression was conducted for six groups of rat hippocampal slices (5–7 weeks old): wild‐type control slices, wild‐type slices treated with miR‐134i, wild‐type slices treated with scrambled miR‐134i, Aβ (1–42)‐treated slices, Aβ (1–42)‐ and miR‐134i‐treated slices and finally Aβ (1–42)‐ and scrambled miR‐134i‐treated slices. Four groups of slices from aged mice hippocampus (16–18 months old): wild‐type aged control, Aβ (1–42)‐treated aged hippocampal slices, Aβ (1–42)‐ and miR‐134i‐treated slices and Aβ (1–42)‐ and scrambled miR‐134i‐treated slices were also collected for RNA isolation and subsequent qRT‐PCR analysis. All slices were collected after strong tetanization (STET) and 4‐hr recording. All hippocampal slices were flash‐frozen in liquid nitrogen and stored at −80°C. Total RNA including small RNAs were extracted from the collected hippocampal slices using miRNeasy Mini Kit according to the manufacturer's instructions and total RNA was quantified using a spectrophotometer (NanoDrop 2000; Thermoscientific).

### 2.4 miRNA quantitative real‐time PCR

Conversion of miRNA to cDNA was performed using the Universal cDNA Synthesis Kit (miRCURY UniRT Starter Kit—Prod No. 203351; Exiqon) according to the manufacturer's instructions. For miRNA quantification, the miRCURY LNATM Universal RT microRNA PCR system (Prod No. 203351; Exiqon) was used in combination with predesigned primers (Prod No. 205989; Exiqon) for hsa‐miR‐134‐5p and miRNA 103a‐3p, an unchanged miRNA as reference gene. miRNA expression was quantified using real‐time PCR system (Model No.7500; Applied Biosystems, Life technologies, Carlsbad, CA, USA). Each sample was measured in duplicates and the expression of miR‐134‐5p was normalized to the expression levels of miR‐103‐3p (according to the manufacturer's instruction). 12–14 slices each from 3 different biological samples were used for miRNA‐134 expression analysis (*n* = 3).

### 2.5 mRNA quantitative real‐time PCR

For mRNA expression analysis, cDNA conversion was carried out using GoScript Reverse Transcription System (Promega, USA). In brief, 2 μg of RNA was subjected to preheating with 2 μl Oligo (dT) at 72°C for 2 min. Reverse transcription was performed at 42°C for 1 hr followed by 95°C for 5 min. Further, StepOne Plus Real‐time PCR system (Applied Biosystems) was used to carry out the qRT‐PCR with Taqman universal PCR master mix (Cat. No. 4,304,437; Thermo Scientific) and TaqMan probes specific for BDNF and CREB. The qRT‐PCR was performed in 96‐well plates with denaturation at 95°C for 10 min, 40 amplification cycles each of 95°C for 15 s and finally 60°C for 1 min. Fold changes of BDNF and CREB gene expressions were calculated according to 2^−ΔΔCt^ method (Livak & Schmittgen, 2001). Each sample was measured in duplicates and was normalized to the internal control GAPDH. 12–14 slices each from four different biological samples were used for each gene expression analysis (*n* = 4).

### 2.6 Western blot analysis

Hippocampal slices were collected from four groups, wild‐type control, Aβ (1–42)‐treated slices, Aβ (1–42)‐ and miR‐134i‐treated slices and Aβ (1–42)‐ and scrambled miR‐134i‐treated slices. The slices from all four groups were collected after STET and 4‐hr recording. All hippocampal slices were flash‐frozen in liquid nitrogen and stored at −80°C. Total protein was extracted from the hippocampal slices using the T‐PER Tissue Protein Extraction Kit (Prod#78510; Thermo Fish Scientific Inc) and HaltTM Protease Inhibitor Cocktail Kit (Prod#78410; Thermo Fish Scientific Inc). Bradford assay was used to quantify the protein level in the samples (Cat. No.500–0007, Bio‐Rad). 20 mg of protein extracts were separated on 10% SDS‐polyacrylamide gels and transferred to polyvinylidene difluoride transfer membranes. The membranes were blocked with 5% nonfat dry milk and incubated with primary antibodies over night at 4°C. The primary antibodies used are as follows: rabbit anti‐CREB (1:500, Cat.No#9197; Cell Signalling), rabbit anti‐p‐CREB (1:500, Cat No# 9198; Cell Signalling), rabbit anti‐BDNF (1:1,000, Cat.No.#ab108319; Ab Cam) and mouse anti‐tubulin monoclonal antibody (Cat No: T9026; Sigma‐Aldrich). Membranes were incubated with the horseradish peroxidase‐conjugated secondary antibody the next day (1:3,000, Cat. No. #170‐6515; Bio‐Rad) for 1 hr. The immunoproducts were detected using a chemiluminescence detection system according to the manufacturer's instructions (Cat. No.# 34,580; Supersignal West Pico Plus Chemiluminescent Substrate, Pierce Biotechnology) and developed on a film. Image J software was used to quantify the optical density of each protein band. Each lane of protein band density was normalized with the corresponding α‐tubulin protein density.

### 2.7 Statistics

All data are represented as mean ± *SEM*. The average values of the slope function of the field EPSP (millivolts per millisecond) expressed as percentages of average baseline values per time point were analysed using the Wilcoxon signed‐rank test (Wilcox's test) when comparing within one group and the Mann–Whitney *U* test (*U* test) when data were compared between groups. The nonparametric test was used because of the normality violation at small sample size.

Data used for statistical analysis for qRT–PCR and Western blot were derived from three to four independent experiments and presented as mean ± *SD*. Statistical significance was evaluated either by the Student's *t* test (for one to one comparison) or one‐way ANOVA (for multiple comparisons). Results were considered as significant at *p* < .05. The statistical analyses were performed using the Prism software (GraphPad).

## 3 RESULTS

### 3.1 Aβ (1–42) oligomer treatment impairs late LTP but not early LTP in acute hippocampal slices

Previous studies reported that Aβ (1–42) treatment results in late‐LTP impairment in CA1 pyramidal neurons **(**Sharma et al., 2017, Krishna et al., 2016). We reproduced this result in the current study. In brief, acute hippocampal rat slices were pre‐incubated with Aβ (1–42) for 3 hr and strong high‐frequency stimulation (STET: 100 Hz, 100 pulses at 0’, 10’ and 20’) applied to synaptic input S1, resulted in an LTP that gradually declined to baseline (Figure 1b, blue closed circles), whereas wild‐type slices showed long‐lasting late LTP lasting for 240 min (Figure1b, red closed circles) which is similar to previous reports. Statistically significant potentiation was observed in S1 after STET induction in control slices and was maintained till the end of the recording period of 240 min (Figure 1b, red closed circles; *n* = 7; Wilcox's test, *p* = .03, *U* test, *p* = .002). For Aβ (1–42) pretreated slices, the LTP was statistically significant until 120 min (Figure 1b, blue closed circles, Wilcox's test, *p* = .03, *U* test, *p* = .002). Control potentials in S2 were stable for both experiments throughout the recordings (Figure 1b, red open circles, blue open circles). Application of weak tetanization (WTET: 100 Hz, 21 pulses, single burst, 0.2 ms pulse duration) resulted in a transient form of LTP lasting 2–3 hr in both control and Aβ (1–42) pretreated slices (Figure 1c, red closed circles, blue closed circles), similar to earlier reports (Krishna et al., 2016; Sharma et al., 2017). The early LTP in control slices stayed statistically significant up to 60 min (Figure 1c, red closed circles, Wilcox's test, *p* = .03) and 120 min (*U* test, *p* = .004) and 90 min (blue closed circles, Wilcox's test, *p* = .03) and 120 min (*U* test, *p* = .004) in Aβ (1–42) pretreated slices, after which it reached baseline within 3 hr (Figure 1c). The control potential S2 was stable till the end of the recordings (Figure 1c, red open circles, blue open circles).

### 3.2 Aβ (1–42) treatment elevates the expression of miR‐134‐5p in rat hippocampal slices

A recent miRNA profiling study has revealed an upregulation of miR‐134 expression in human AD patient brain samples (Moradifard et al., 2018). In the present study, qRT‐PCR analysis confirmed that miR‐134‐5p expression was significantly increased (about 3.5 fold) in Aβ (1–42)‐treated rat hippocampal slices where plasticity is known to be impaired, when compared to the control slices (Figure 1d, student's *t* test, *p* = .0008).

In order to understand the functional role of miR‐134‐5p in AD pathology, miR‐134‐5p knockdown analysis was carried out using miR‐134‐5p antagomir, referred to as miR‐134‐5p inhibitor (miR‐134i). A nonspecific scrambled inhibitor of miR‐134‐5p was used as negative control. In brief, control slices and Aβ (1–42)‐treated slices were transfected with miR‐134i (1µM) and scrambled miR‐134i (SCi) for 3 hr (Figure 1e and 1f). qRT‐PCR analysis showed that miR‐134‐5p expression was reduced significantly after treatment with miR‐134i, when compared to the scrambled inhibitor in both wild‐type and Aβ (1–42)‐treated slices (Figure 1e, student's *t* test, *p* = .004 and Figure 1f, student's *t* test, *p* = .006), indicating effective knockdown of miR‐134‐5p in hippocampal slices. After the inhibition of miR‐134‐5p expression in the Aβ (1–42)‐treated slices using miR‐134i, we tested whether plasticity impairments mediated by Aβ (1–42) can be reversed by inhibition of miR‐134‐5p expression.

### 3.3 Knockdown of miR‐134‐5p rescues Aβ (1–42)‐induced deficit in late LTP

**Figure 2:**
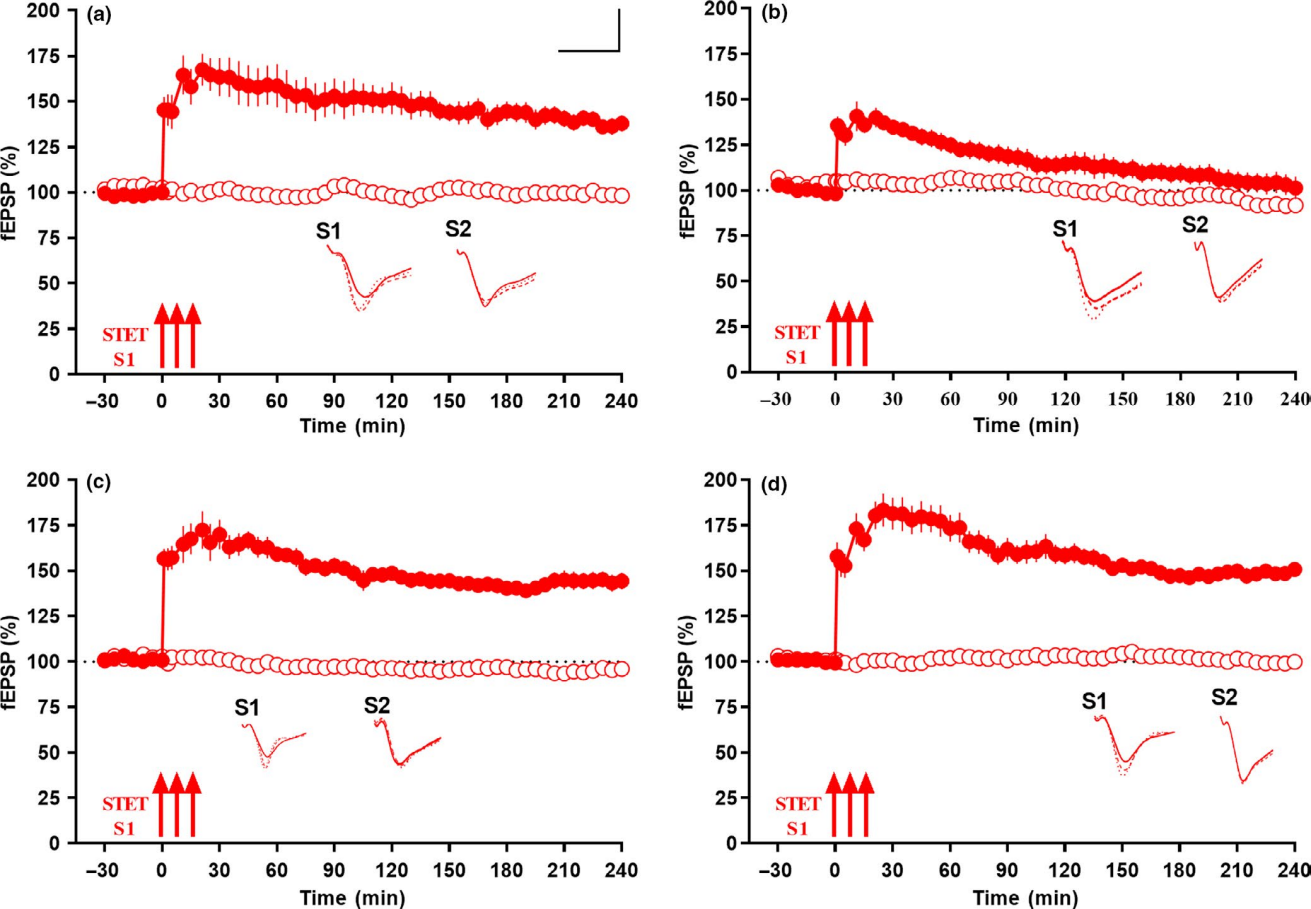
miR‐134 knockdown by miR‐134i rescues late LTP in Aβ (1–42)‐induced rat hippocampal slices: (a) Late LTP was maintained for 4 hr when a strong tetanization (STET) was applied to S1 (red closed circles) while control baseline potentials in S2 (red open circles) remained stable in miR‐134i (1 μM) and Aβ (1–42) (200 nM) pretreated slices (*n* = 7). (b) Late LTP by STET in S1 (red closed circles) in scrambled inhibitor (SCi) (1 μM) and Aβ (1–42) (200 nM) pretreated slices was impaired while basal potential in S2 (red open circles) remained stable throughout the recording period (*n* = 7). (c) Late LTP was maintained for 4 hr when a strong tetanization (STET) was applied to S1 (red closed circles) while control baseline potentials in S2 (red open circles) remained stable in miR‐134i (1 μM) pretreated wild‐type slices (*n* = 6). (d) Late LTP by STET in S1 (red closed circles) in scrambled inhibitor (SCi) (1 μM) pretreated wild‐type slices maintained for 4 hr while basal potential in S2 (red open circles) remained stable throughout the recording period (*n* = 8). All symbols/traces are as in Figure 1. Calibration bar for all analog sweeps: vertical: 3 mV; horizontal: 5 ms

Aβ (1–42) treatment to hippocampal rat slices have been known to affect long‐term plasticity (late LTP) and associativity such as synaptic tagging and capture (STC) (Jiang et al., 2015; Krishna et al., 2016; Lei et al., 2016; Ma et al., 2014; Quenon, de Xivry, Hanseeuw, & Ivanoiu, 2015; Sharma et al., 2017). Since the impaired synaptic plasticity was associated with the upregulation of miR‐134‐5p expression in rat hippocampal slices, we investigated if inhibition of miR‐134‐5p expression using miR‐134i could rescue late LTP in Aβ (1–42)‐treated hippocampal slices. In the first set of experiments, Aβ (1–42) (200 nM) and miR‐134i (1 µM) were bath applied to hippocampal slices for 3 hr and a stable baseline of 30 min was recorded from the CA1 region (for more details about drug application, see experimental design depicted in Figure S1 A). Strong high‐frequency stimulation (STET, 100 Hz, 100 pulses at 0’, 10’ and 20’) applied to synaptic input S1 resulted in long‐lasting late LTP lasting at least 240 min (Figure 2a). Statistically significant potentiation was observed in S1 after STET application and was maintained till the end of the recording period of 240 min (Figure 2a, red closed circles; *n* = 7; Wilcox's test, *p* = .01, *U* test, *p* = .0006). As a control, the same experiments were repeated using the scrambled inhibitor, which resulted in an LTP that gradually declined to baseline (Figure 2b, red closed circles; *n* = 7). The LTP was statistically significant until 100 min (Figure 2b, Wilcox's test, *p* = .04) and up to 85 min (*U* test, *p* = .02). In both cases (Figure 2a and 2b), baseline potentials from synaptic input S2 (red open circles) stayed relatively stable till the end of the recording period implying that the addition of miR‐134i or scrambled inhibitor did not affect basal synaptic responses. No significant changes were observed in this input (Figure 2a, Wilcox's test, *p* = .37; Figure 2b, Wilcox's test, *p* = .07). Overall, these results indicate that miR‐134i can re‐establish late LTP, which was impaired by Aβ (1–42), in the hippocampal CA1 region.

In another set of experiments with wild‐type slices alone, miR‐134i (1 µM) or scrambled inhibitor (1 µM) were bath applied to these slices for 3 hr and a stable baseline of 30 min was recorded from the CA1 region (Figure 2c and Figure 2d). STET applied to synaptic input S1 in miR‐134i‐treated slices resulted in long‐lasting late‐LTP lasting for 240 min (Figure 2c, red closed circles). Statistically significant potentiation was observed in S1 after STET application and was maintained till the end of the recording period of 240 min (Figure 2c, red closed circles; *n* = 7; Wilcox's test, *p* = .03, *U* test, *p* = .001). In scrambled miR‐134 inhibitor‐treated slices, significant potentiation was observed in S1 after late LTP induced by STET and maintained till the end of the recording (Figure 2d, red closed circles; *n* = 8; Wilcox's test, *p* = .007, *U* test, *p* = .0002). In both cases (Figure 2c and 2d), baseline potentials from synaptic input S2 (red open circles) were stable till the end of the recording period. We did not see a significant increase in potentiation percentage of late‐LTP expression in wild‐type slices treated with miR‐134i compared to wild‐type slices treated with scrambled inhibitor. This suggests that miR‐134 inhibitor‐mediated rescue of late LTP was specific to Aβ‐treated slices.

We have also checked the early phase of LTP (early LTP) using WTET in miR‐134i‐treated wild‐type slices and it showed that early LTP was intact in wild‐type slices treated with miR‐134i (Figure S3 B, red closed circles, *n* = 5). WTET application to S1 (red closed circles) at the 60th minute after a stable baseline of 30 min led to a potentiation that gradually declined to baseline within 3 hr (Figure S3 B). The early‐LTP potentiation was statistically significant up to 60 min (Wilcox's test, *p* = .03) and 80 min (*U* test, *p* = .007), after which it reached baseline within the 3‐hr recording (Figure S3 B). Control potentials in S2 (red open circles) remained stable at baseline throughout the recording.

### 3.4 Rescue of late LTP, by inhibiting miR‐134‐5p expression, is protein synthesis‐ and NMDAR‐dependent

As a prerequisite to study associative plasticity such as synaptic tagging and capture (STC), we tested whether the late LTP expressed due to miR‐134 inhibition was maintained by newly synthesized PRPs. To test this, we used two structurally distinct protein synthesis inhibitors, anisomycin and emetine, which were bath applied for 1.5 hr, after recording a stable baseline of 30 min (Figure S2 A and B). Late‐LTP induction by STET 30 min after drug application resulted in a decremental LTP in both cases (Figure S2 A and B, red closed circles). The experiments in which anisomycin (25 µM) was bath applied showed statistically significant potentiation after STET (Figure S2 A, red closed circles) until 115 min (Wilcox's test, *p* = .03) and up to 130 min (*U* test, *p* = .004), whereas in case of emetine (20 µM, Figure S2 B), potentiation after the induction of late LTP stayed statistically significant up to 125 min (red closed circles, Wilcox's test, *p* = .04) and 130 min (*U* test, *p* = .03). It has been shown earlier that activation of NMDA receptor is critical for the setting of synaptic tags (O’Carroll & Morris, 2004). To test the activation of NMDA receptor during the induction of late LTP in miR‐134 inhibited hippocampal neurons, the receptor antagonist AP5 (50 µM) was bath applied for 45 min before and after the induction of late LTP by STET in S1 (Figure S2 C). No potentiation was observed in S1 (red closed circles) and both S1 and S2 (red closed and red open circles) remained at baseline level throughout the entire recording period of 4 hr (Figure S2 C; Wilcox's test, *p* = .46; *U* test, *p* = .68). Control input in S2 in all experiments (Figure S2 A–B, red open circles) stayed relatively stable. In brief, protein synthesis and NMDA receptor activity were essential for the reinstatement of synaptic plasticity in Aβ (1–42)‐treated hippocampal slices where miR‐134‐5p was inhibited.

### 3.5 Knockdown of miR‐134‐5p rescues Aβ (1–42)‐induced impairment of synaptic tagging and capture in rat hippocampal slices

**Figure 3:**
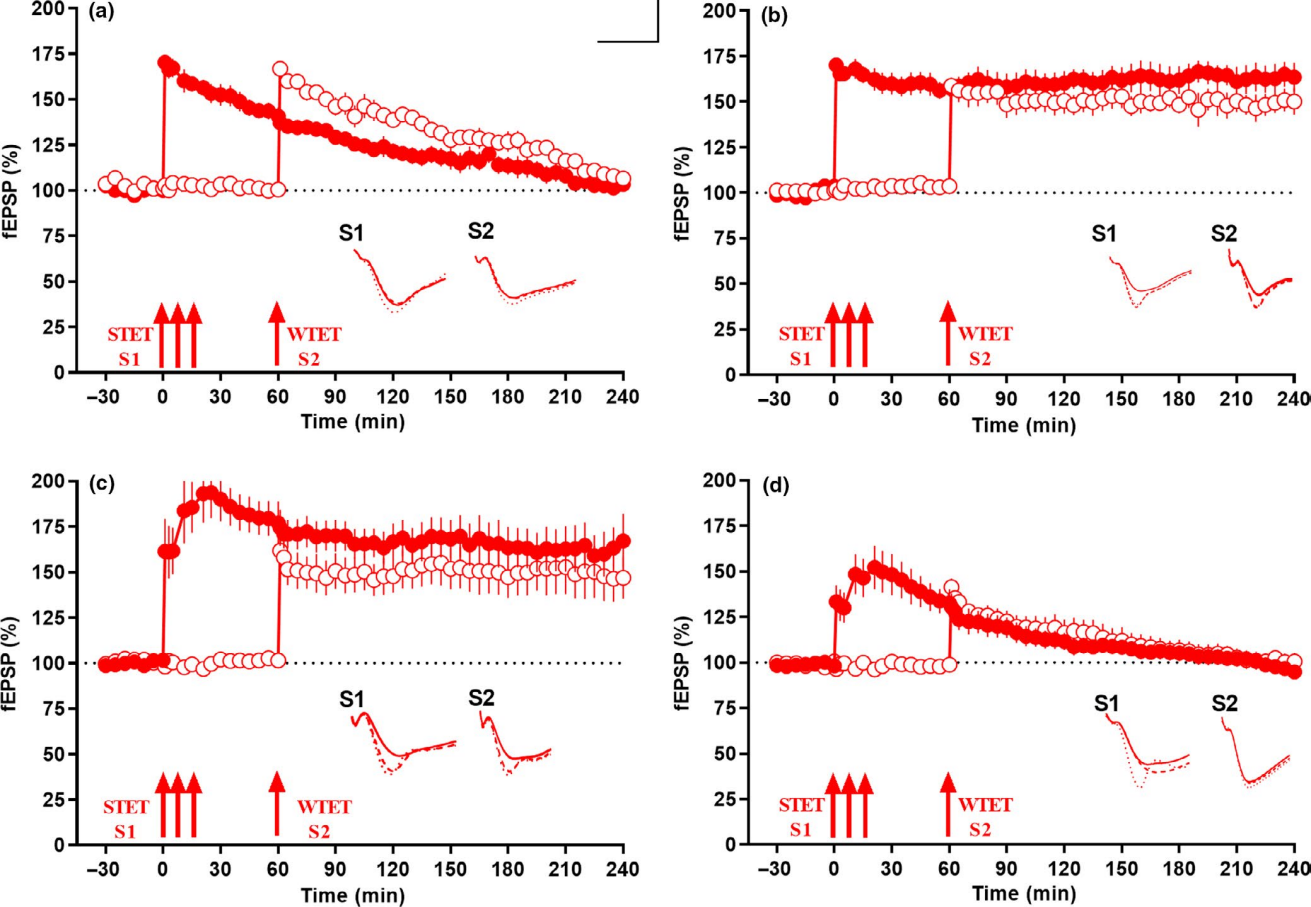
miR‐134 knockdown ameliorates Aβ (1–42)‐induced deficit in synaptic tagging and capture (STC). (a) Strong before weak paradigm in which STET applied in S1 (red closed circles) at 0 min to induce late LTP and WTET applied in S2 to induce early LTP (red open circles) at 60 min in Aβ(1–42) (200 nM) pretreated slices, potentiation in both synaptic inputs returned to baseline within 4 hr. No STC was observed in this condition while in (b), the same experimental design in control slices showed STC. Early LTP in S2 was transformed to late LTP by capturing PRPs from S1. (c) STET applied in S1 (red closed circles) at 0 min and WTET applied in S2 (red open circles) at 60 min in miR‐134i (1 μM) and Aβ(1–42)(200 nM) pretreated slices showed long‐lasting potentiation for 4 hr resulted in late LTP in both the synaptic inputs, thereby expressing STC (*n* = 7). (d) STET applied in S1 (red closed circles) at 0 min and WTET applied in S2 (red open circles) at 60 min in SCi (1 μM) and Aβ(1–42) (200 nM) pretreated slices both returned to baseline within 4 hr and failed to express late LTP in both the inputs S1 and S2 (*n* = 6). All data presented as mean ± *SEM*. The three arrows represent strong tetanization (STET) applied for inducing late LTP. Single arrow represents weak tetanization (WTET) applied for inducing early LTP. Insets in each graph represent typical fEPSP traces recorded 15 min before (continuous line), 30 min after (dotted line) and 240 min after (broken line) the induction of LTP. Calibration bar for all analog sweeps: vertical: 3 mV; horizontal: 5 ms

Exogenous application of Aβ (1–42) oligomer impairs synaptic tagging and capture (STC) in hippocampal slices (Krishna et al., 2016; Sharma et al., 2017). In order to confirm the effect of exogenous application of Aβ (1–42) oligomer on STC, we used the “strong before weak” paradigm, in which STET was delivered to synaptic input S1 prior to WTET in S2 with an interval of 60 min (Frey & Morris, 1997, 1998). After a stable baseline of 30 min, STET was applied to S1 (Figure 3a, red closed circles) followed by WTET in S2 at the 60th min (red open circles). The slices treated with Aβ failed to express STC (Figure 3a, red closed and red open circles) unlike the control, where both S1 and S2 expressed a significant potentiation for 4 hr (Figure 3b, red closed and red open circles). For slices pretreated with Aβ (1–42), in input S1 (red closed circles), STET at 0 min resulted in a potentiation that was significant until 120 min (Wilcox's test, *p* = .03) and WTET at 60 min in input S2 (red open circles) resulted in a potentiation that was significant until 120 min (Wilcox's test, *p* = .03), whereas in wild‐type slices, significant potentiation was observed in both S1 (Figure 3b, red closed circles, Wilcox's test, *p* = .03) and S2 (Figure 3b, red open circles, Wilcox's test, *p* = .03), thus expressing STC.

Next, we tested the effect of miR‐134i co‐applied with Aβ (1–42) oligomer on STC using the same paradigm where we induced STET in S1 (red closed circles) at 0 min after a stable 30 min baseline, followed by WTET in synaptic input S2 (red open circles) at 60 min (Figure 3c; *n* = 7). Significant potentiation was observed in both S1 (red closed circles, Wilcox's test, *p* = .01) and S2 (red open circles, Wilcox's test, *p* = .01), thereby expressing STC. In a control experiment, the hippocampal slices treated with scrambled miR‐134‐5p inhibitor failed to express STC as both inputs decayed to baseline by 150–180 min (Figure 3d). Potentiation in synaptic input S1 (red closed circles) was significant until 100 min (Wilcox's test, *p* = .03) and WTET at 60 min in input S2 (red open circles) resulted in potentiation that was significant until 120 min (Wilcox's test, *p* = .03). Our results indicated that the STC expressed after miR‐134i treatment in Aβ (1–42)‐treated hippocampal slices was brought about by specific knockdown of miR‐134 and the subsequent increase in newly synthesized proteins.

We confirmed that the conversion of early LTP to late LTP was due to tagging and not due to the reinforcement effect of miR‐134 inhibitor as induction using WTET in miR‐134 inhibited Aβ (1–42)‐treated slices resulted only in an early LTP (Figure S3 A; *n* = 6). WTET application to S1 (red closed circles) at the 60th minute after a stable baseline of 30 min led to a potentiation that gradually declined to baseline within 3 hr (Figure S3 A, red closed circles). The early‐LTP potentiation was statistically significant up to 150 min (Wilcox's test, *p* = .03) and 155 min (*U* test, *p* = .04), after which it reached baseline within the 3 hr recording (Figure S3 A, red closed circles). Control potential S2 (red open circles) remained stable at baseline throughout the recording and any changes observed were statistically insignificant (Wilcox's test, *p* = .43).

### 3.6 Knockdown of miR‐134‐5p in Aβ (1–42)‐treated hippocampal slices upregulates mRNA and protein expression levels of CREB‐1 and BDNF

**Figure 4:**
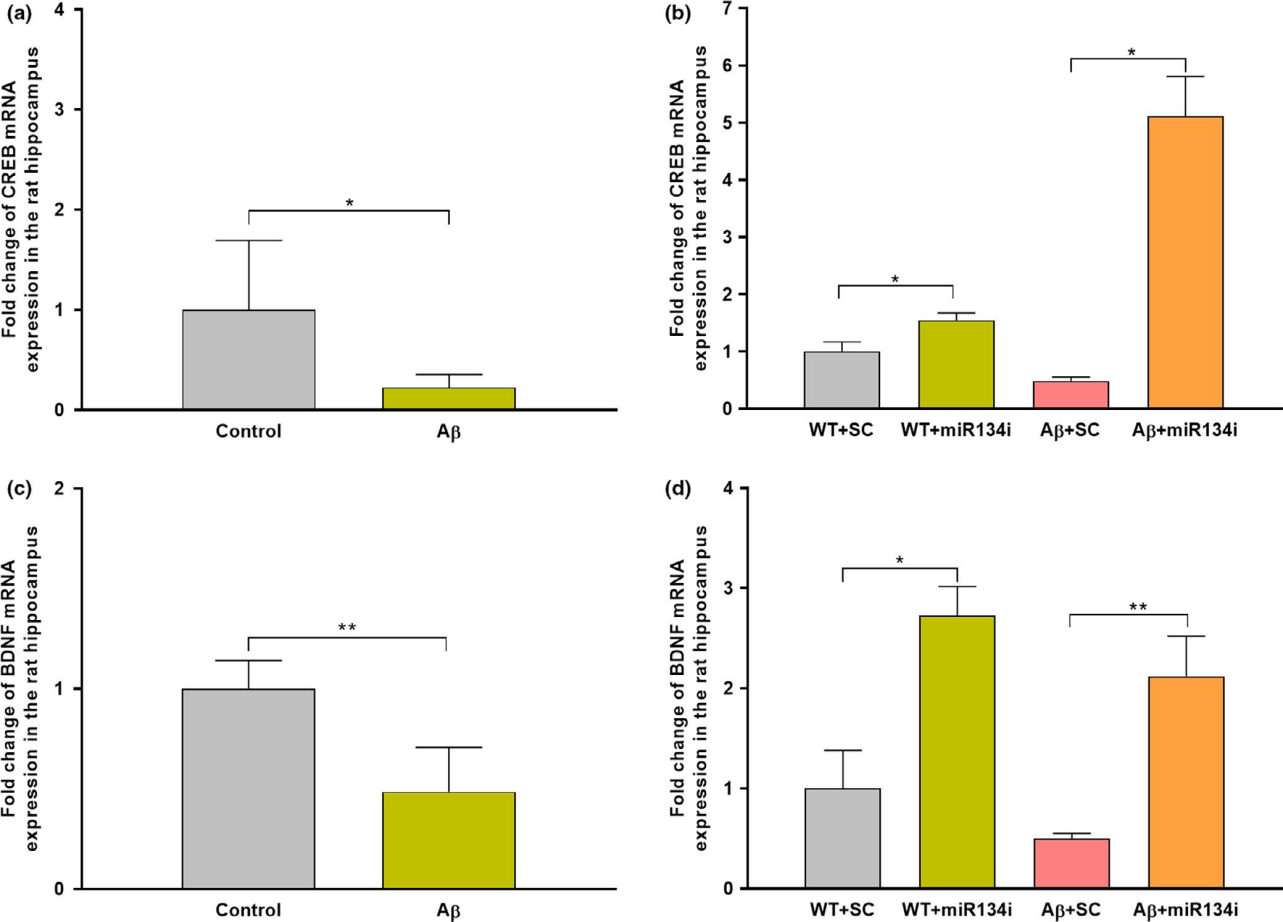
miR‐134 knockdown elevates CREB and BDNF mRNA expression in Aβ(1–42)‐treated rat hippocampus: (a) qRT‐PCR analysis showing a significant reduction of CREB‐1 mRNA expression in Aβ(1–42)‐treated hippocampal slices. Each sample was measured in duplicates and normalized to the internal control GAPDH. Significant differences between the two groups, Control versus Aβ, are indicated by *p ˂ .05 (student's *t* test, 12 slices each from 4 different biological samples, *n* = 4). (b) qRT‐PCR analysis showing a significant increase in CREB‐1 mRNA expression in miR‐134 knockdown wild‐type slices treated with or without Aβ(1–42) compared to scrambled miR‐134 inhibitor‐treated wild‐type slices with or without Aβ(1–42). Each sample was measured in duplicates and normalized to the internal control GAPDH. Significant differences between the groups: WT + SCi versus WT + miR‐134i and SCi + Aβ versus miR‐134i + Aβ are indicated by *p ˂ .05, (one‐way ANOVA, 12 slices each from 4 different biological samples, *n* = 4). (c) qRT‐PCR analysis showing a significant decrease of BDNF mRNA expression in Aβ(1–42)‐treated rat hippocampal slices. Each sample was measured in duplicates and normalized to the internal control GAPDH. Significant differences between the two groups Control versus Aβ are indicated by **p ˂ .01 (student's *t* test, 12 slices each from 4 different biological samples, *n* = 4). (d) miR‐134 knockdown using miR‐134i in wild‐type slices treated with or without Aβ (1–42) significantly elevated BDNF mRNA levels when compared to scrambled miR‐134 inhibitor‐treated wild‐type slices co‐treated with or without Aβ(1–42). Each sample was measured in duplicates and normalized to the internal control GAPDH. Significant differences between the groups: WT + SCi versus WT + miR‐134i and SCi + Aβ versus miR‐134i + Aβ are indicated by *p ˂ .05, **p ˂ .01 (one‐way ANOVA, 12 slices each from 4 different biological samples, *n* = 4)

CREB and BDNF, the two plasticity proteins crucial for the formation and maintenance of long‐term plasticity and associativity (Caracciolo et al., 2018; Korte et al., 1995; Sajikumar & Korte, 2011), are post‐transcriptionally regulated by miR‐134 via a Sirtuin1‐mediated mechanism (Gao et al., 2010). Furthermore, TargetScan analysis revealed that CREB‐1 is one of the direct targets of miR‐134‐5p (data not shown). The qRT‐PCR analysis showed that CREB‐1 and BDNF mRNA expression levels were significantly decreased in Aβ (1–42)‐treated hippocampal slices when compared to wild‐type control slices (Figure 4a, student's *t* test, *p* = .03 and 4C, student's *t* test, *p* = .006). However, the expression levels of both CREB‐1 and BDNF were increased significantly in hippocampal slices co‐treated with miR‐134i and Aβ (1–42), when compared to the respective scrambled miRNA 134‐5p inhibitor slices co‐treated with Aβ (1–42) (Figure 4b, one‐way ANOVA, *p* = .01 and 4D, one‐way ANOVA, *p* = .003). The wild‐type slices treated with miR‐134i alone also showed an increase in the mRNA levels of CREB‐1 and BDNF when compared to scrambled inhibitor‐treated groups (Figure 4b, one‐way ANOVA, *p* = .03 and 4D, one‐way ANOVA, *p* = .03). However, we observed that the effect of miR‐134‐5p on CREB and BDNF expression in untreated rat hippocampal slices was less robust than that in Aβ (1–42)‐treated slices.

**Figure 5:**
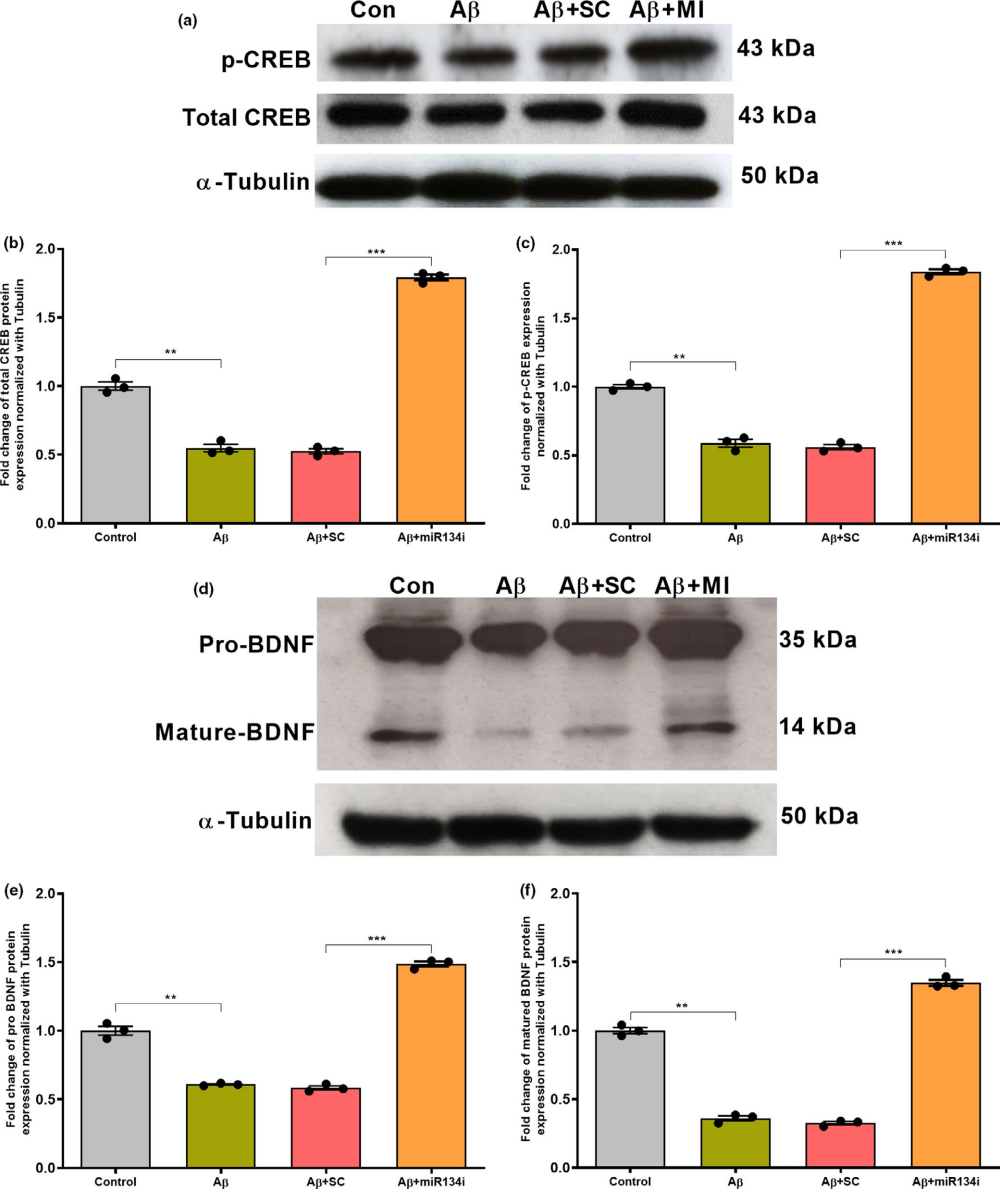
(a–c) miR‐134 knockdown elevates total CREB and p‐CREB levels in rat hippocampal slices: Western blot analysis showing a significant reduction in total CREB and p‐CREB levels in Aβ(1–42)‐treated young rat hippocampal slices when compared to that of wild‐type control (a–c). However, a significant increase in total CREB and p‐CREB expression was detected in miR‐134 knockdown Aβ(1–42)‐treated rat hippocampal slices when compared to the respective scrambled inhibitor (SCi)‐treated group (a–c). Total CREB (43 kDa), p‐CREB (43 kDa) and α‐Tubulin (50 kDa) immunoreactive bands are shown (a). The data are normalized to respective tubulin. Significant differences between the groups (Control versus Aβ and SCi + Aβ versus miR‐134i + Aβ are indicated by **p ˂ .01, **p ˂ .001, (one‐way ANOVA, 12 slices each from 4 different biological samples, *n* = 4). (d–f) miR‐134 knockdown increases pro‐ and mature‐BDNF level: Western blot analysis shows that both pro‐ and mature‐BDNF protein levels were reduced in Aβ (1–42)‐treated young rat hippocampus and SCi + Aβ (1–42)‐treated young rat hippocampus when compared to wild‐type control hippocampus (d–f). However, a significant increase in both pro‐ and mature‐BDNF expression was observed in miR‐134 knockdown Aβ (1–42)‐treated hippocampal slices when compared to the respective scrambled inhibitor‐treated groups (d–f). Pro‐BDNF (35 kDa), mature‐BDNF (14 kDa) and α‐Tubulin (50 kDa) immunoreactive bands are shown (d). The data are normalized to respective tubulin. Significant differences between the groups: Control versus Aβ, SCi + Aβ versus miR‐134i + Aβ are indicated by **p ˂ .01, ***p ˂ .001 (one‐way ANOVA, 12 slices each from 4 different biological samples, *n* = 4)

The expression pattern was further confirmed by Western blot analysis which showed that both CREB‐1 and BDNF (pro and mature) protein expression levels were significantly reduced in Aβ (1–42)‐treated hippocampal slices (Figure 5a‐b, one‐way ANOVA, *p* = .008 and 5D‐F, one‐way ANOVA, *p* = .008, *p* = .002). However, miR‐134 knockdown by miR‐134i significantly increased the protein expression levels of CREB‐1 and pro and mature BDNF in Aβ (1–42)‐treated slices (Figure 5a‐b, one‐way ANOVA, *p* = .0009 and 5D‐F, one‐way ANOVA, *p* = .0005, *p* = .0003). Interestingly, we observed that the phosphorylation level of CREB‐1 was also increased in hippocampal slices co‐treated with miR‐134i and Aβ (1–42) (Figure 5a, 5, one‐way ANOVA, *p* = .0007), indicating a novel miR‐134‐CREB‐BDNF mechanism in rescuing late‐LTP and STC impairment in Aβ‐induced AD condition.

### 3.7 Knockdown of miR‐134‐5p expression in aged mice hippocampal slices treated with Aβ (1–42) rescued late‐LTP and elevated CREB‐1 and BDNF expression

We have checked the expression levels of miR‐134‐5p and measured late‐LTP expression from aged mice (16–18 months old) hippocampal slices treated with or without Aβ (1–42) and miR‐134i for 3 hr, similar to experiments in Wistar rat slices. qRT‐PCR analysis showed that miR‐134‐5p expression was elevated in aged mice hippocampal slices treated with Aβ (1–42) compared to control aged mice slices (Figure S4 A, student's *t* test, *p* = .04). miR‐134‐5p knockdown in aged mice hippocampal slices was confirmed using qRT‐PCR analysis which indicated that miR‐134‐5p levels were significantly reduced in Aβ (1–42) and miR‐134i co‐treated slices compared to Aβ (1–42) and scrambled inhibitor co‐treated slices (Figure S4 B, student's *t* test, *p* = .03). The mRNA expression levels of CREB‐1 and BDNF were analysed using qRT‐PCR which showed that CREB‐1 and BDNF mRNA expression levels were reduced in aged slices treated with Aβ (1–42) (Figure S4 C, one‐way ANOVA, *p* = .001 and Figure S4 D, one‐way ANOVA, *p* = .04). However, knockdown of miR‐134‐5p elevated the mRNA levels of CREB‐1 and BDNF in these slices (Figure S4 C, one‐way ANOVA, *p* = .003) and Figure S4 D, one‐way ANOVA, *p* = .0002).

Late LTP induced by STET to synaptic input S1 in aged mice (16–18 months old) hippocampal slices resulted in a potentiation which was comparatively less (Figure S5 A, red closed circles) than that observed in young wild‐type mice (5 weeks old) (Figure S5 E, red closed circles), an observation similar to our earlier reports (Sharma, Shetty, Arumugam, & Sajikumar, 2015). The potentiation was statistically significant till 240 min for both aged mice (Figure S5 A, red closed circles; *n* = 5; Wilcox's test, *p* = .03, *U* test, *p* = .002) and young mice slices (Figure S5 E, red closed circles; *n* = 5; Wilcox's test, *p* = .03, *U* test, *p* = .007). Control potentials in S2 (Figure S5 A and Figure S5 E, red open circles) were stable at baseline throughout the recording. Acute aged mice hippocampal slices were pre‐incubated with Aβ (1–42) for 3 hr and late LTP induced by STET resulted in an impaired late LTP (Figure S5 B, red closed circles). Statistically significant potentiation was observed in S1 after STET application (Figure S5 B, red closed circles, *n* = 6) until 105 min (Wilcox's test, *p* = .03) and up to 145 min (*U* test, *p* = .002) after which it reached baseline within the 3‐hr recording. Similar to our observations in Wistar rat hippocampal slices, knockdown of miR‐134‐5p using miR‐134i rescued the late‐LTP impairment in aged mice slices treated with Aβ (1–42) (Figure S5 C, red closed circles). The potentiation in S1 after STET was statistically significant till the end of the recordings (Figure S5 C, red closed circles, *n* = 8, Wilcox's test, *p* = .007, *U* test, *p* = .0002). Scrambled miR‐134 inhibitor treatment failed to rescue late LTP in the Aβ (1–42)‐treated aged mice slices (Figure S5 D, red closed circles, *n* = 6). The potentiation was significantly different until 60 min (Wilcox's test, *p* = .03) and up to 85 min (*U* test, *p* = .002) (Figure S5 C, red closed circles). Control potentials in S2 (red open circles) were stable at baseline throughout the recording for all experiments indicating that the addition of Aβ (1–42) and miR‐134i or scrambled inhibitor did not affect basal synaptic responses (Figure S5 B‐D, red open circles). Overall, the results from aged mice and young Wistar rat hippocampal slices treated with Aβ (1–42) were similar. miR‐134‐5p inhibition rescued late‐LTP and molecular expression of CREB‐1 and BDNF in aged mice and young Wistar rat slices, both treated with Aβ (1–42).

## 4 DISCUSSION

Alzheimer's disease (AD) is an age‐related neurodegenerative disorder, characterized by the loss of synaptic connections and impairment in synaptic plasticity (Selkoe, 2002). Several microRNAs (miRNAs) have been shown to mediate these plasticity changes in AD (Cogswell et al., 2008; Cohen, Lee, Chen, Li, & Fields, 2011; Müller, Kuiperij, Claassen, Küsters, & Verbeek, 2014). In fact, several miRNA profiling studies have shown that many miRNAs are dysregulated in human AD brain (Cogswell et al., 2008; Moradifard et al., 2018; Nunez‐Iglesias et al., 2010) and this dysregulation seems to be associated with the plasticity changes in AD (Maes, Chertkow, Wang, & Schipper, 2009; Schonrock et al., 2010). Recently, miR‐134, a brain‐specific miRNA, has been shown to be upregulated in AD patient brain samples (Moradifard et al., 2018). The present study confirms that miR‐134‐5p expression is upregulated in Aβ‐induced AD conditions and inhibiting the expression of miR‐134‐5p rescues late LTP, which is otherwise impaired in Aβ‐induced AD conditions (Krishna et al., 2016; Sharma et al., 2017). These findings suggest that downregulation of miR‐134‐5p expression would be helpful in restoring the plasticity deficit in AD.

Aβ (1–42) treatment disrupts the synthesis of plasticity‐related proteins (PRPs) (Pang & Lu, 2004; Sharma et al., 2017), which are required for the maintenance of the late phase of LTP (Frey, Krug, Reymann, & Matthies, 1988). Restoring the protein synthesis ability of neurons is a possible way to overcome the synaptic deficit in AD. We demonstrate that reinstatement of late LTP by inhibiting miR‐134‐5p is dependent on protein synthesis and NMDAR, hence, representing a physiological correlate of memory. These data show that the upregulation of miR‐134‐5p expression in AD is associated with the downregulation of PRPs, and hence, suggest that inhibition of miR‐134‐5p expression in AD could restore protein synthesis and subsequently the late phase of LTP.

Synaptic associativity such as synaptic tagging and capture (STC), a unique feature of healthy neurons, enables weak memory engram to transform to relatively stable memory engram and thus helps with the formation of long‐term memory (Frey & Morris, 1998; Redondo & Morris, 2011; Sajikumar & Frey, 2004). Various studies have reported the dysregulation of synaptic associativity in AD pathology (Bastin et al., 2014; Jiang et al., 2015; Quenon et al., 2015). It has been reported earlier that STC is highly impaired during aging and in Aβ‐induced AD conditions (Sharma et al., 2017, 2015; Shetty & Sajikumar, 2017). Our data show that downregulation of miR‐134‐5p could rescue Aβ‐induced impairment of STC. STC is primarily characterized by two events: one is the activity‐dependent “tagging” of the synapses and the other is the “capture” of plasticity proteins by the synaptic tags. The impairment of STC in Aβ‐induced AD condition could be due to the disruption of either of these two events. It could be that tag setting may not be affected in Aβ‐treated neurons since we found that early LTP was intact in these neurons. However, the protein synthesis may be disrupted due to the upregulation of miR‐134‐5p which inhibits local protein synthesis at the synapses. We demonstrate that knockdown of miR‐134‐5p re‐instates STC in AD condition, further strengthening our hypothesis that a novel miR‐134‐mediated mechanism is involved in restoring associative plasticity in AD.

Another intriguing observation from our study is that miR‐134‐5p is predicted to target CREB‐1 and post‐transcriptionally regulates the expression of CREB and BDNF in AD conditions. CREB and BDNF are two important plasticity‐related proteins that are involved in synaptic plasticity and memory formation (Caracciolo et al., 2018; Korte et al., 1995; Sajikumar & Korte, 2011). It has been shown previously that miR‐134 controls plasticity and memory via a SIRT1‐mediated regulation of CREB and BDNF (Gao et al., 2010). Furthermore, BDNF mRNA was found to be less abundant in the postmortem hippocampi samples of AD individuals (Phillips et al., 1991), indicating the importance of BDNF in AD pathology. In the current study, downregulation of CREB and BDNF expression in Aβ‐treated rat hippocampal neurons, as reported earlier (Pugazhenthi et al., 2011; Zhang et al., 2015), appears to be attributed to the upregulation of miR‐134‐5p, thereby resulting in the impairment of late LTP and STC. The functional nexus of miR‐134‐5p‐CREB‐BDNF was further confirmed as the inhibition of miR‐134‐5p restores CREB and BDNF expression and thereby late LTP and STC in Aβ‐induced AD condition. Our results also suggest that the rescue of late LTP by inhibiting miR‐134‐5p is specific to Aβ‐induced AD condition. This is attributed to the observed increase in the expression levels of CREB‐1 and BDNF but no significant increase in percentage potentiation of late‐LTP expression, after miR‐134‐5p knockdown in wild‐type slices.

CREB binds to the cAMP Response Element (CRE) on the promoter of the gene coding for RNA polymerase, which in turn regulates expression of other memory‐related genes (Kandel, 2012; Montminy, 1997). The possible upregulation of total protein expression and phosphorylation of CREB, via miR‐134‐5p knockdown, most likely led to the synthesis of various plasticity‐related proteins that impose structural changes in the synapses, support synaptic strengthening, and thereby, maintain late LTP and STC (Korte & Schmitz, 2016) in Aβ‐induced AD condition. The fact that miR‐134‐5p directly targets the mRNA of CREB, a crucial and integrative molecule of memory formation, substantiates the need to further understand the miR‐134‐5p‐mediated signalling mechanism in AD.

We have also shown that inhibition of miR‐134‐5p is capable of increasing BDNF mRNA and protein expression in Aβ‐induced AD conditions. This highlights BDNF as one of the important PRPs involved in the miR‐134‐5p‐mediated rescue of late LTP and STC in Aβ‐induced AD condition. It has been reported earlier that phosphorylation of CREB by Ca^2+^ influx in postsynaptic neurons leads to the binding of CREB to CRE on the BDNF gene, activating BDNF transcription (Tao, Finkbeiner, Arnold, Shaywitz, & Greenberg, 1998). This supports our assumption that miR‐134‐5p knockdown upregulates CREB mRNA, resulting in increased CREB protein expression level and CREB phosphorylation, eventually resulting in elevated BDNF gene transcription.

One of the greatest known risk factors for AD is advanced age (Alzheimer’s Association, 2015; Guerreiro & Bras, 2015; Naj et al., 2014). It was seen that miR‐134‐5p inhibition rescues late‐LTP and molecular expression of CREB‐1 and BDNF in aged mice Aβ‐treated hippocampal slices. This further confirms the unprecedented role of miR‐134‐5p‐CREB‐BDNF in mediating plasticity changes in Aβ‐induced AD conditions. Our future studies will delineate the functional role of miR‐134‐5p in AD hippocampal pyramidal neurons by overexpressing miR‐134‐5p using miR‐134‐5p mimics.

Together, our findings suggest that miR‐134‐5p mediates the plasticity deficit in Aβ‐induced AD condition by post‐transcriptionally regulating the expression of CREB and BDNF. Downregulating miR‐134‐5p expression in AD restores late LTP and STC via modulating CREB and BDNF signalling. These results highlight a novel miR‐134‐CREB‐BDNF‐mediated mechanism in regulating synaptic plasticity and associativity in the AD hippocampus and miR‐134‐5p can be proposed as a potential target for the diagnosis of AD and the development of appropriate therapeutic agents for AD.

## CONFLICT OF INTEREST

The authors declare that the research was conducted in the absence of any commercial or financial relationships that could be construed as a potential conflict of interest.
