# Supplementary figures and images for: MicroRNA‐134‐5p inhibition rescues long‐term plasticity and synaptic tagging/capture in an Aβ(1–42)‐induced model of Alzheimer’s disease
Source: Aging Cell. 2019 Oct 17;19(1):e13046. doi: 10.1111/acel.13046 (PMC6974725; doi:10.1111/acel.13046)

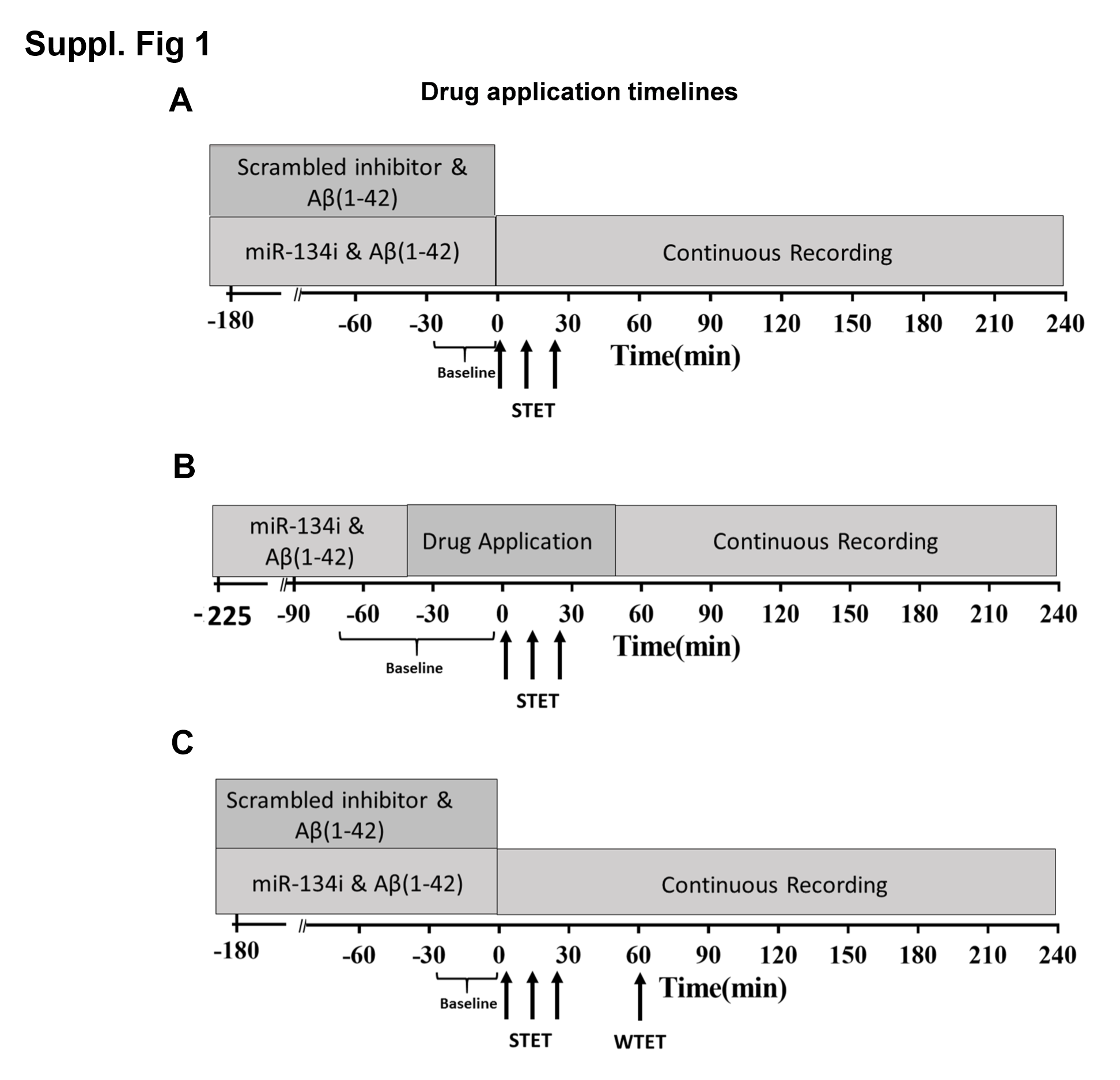

Supplement: Supplementary file 1 [file ACEL-19-e13046-s001.tif]

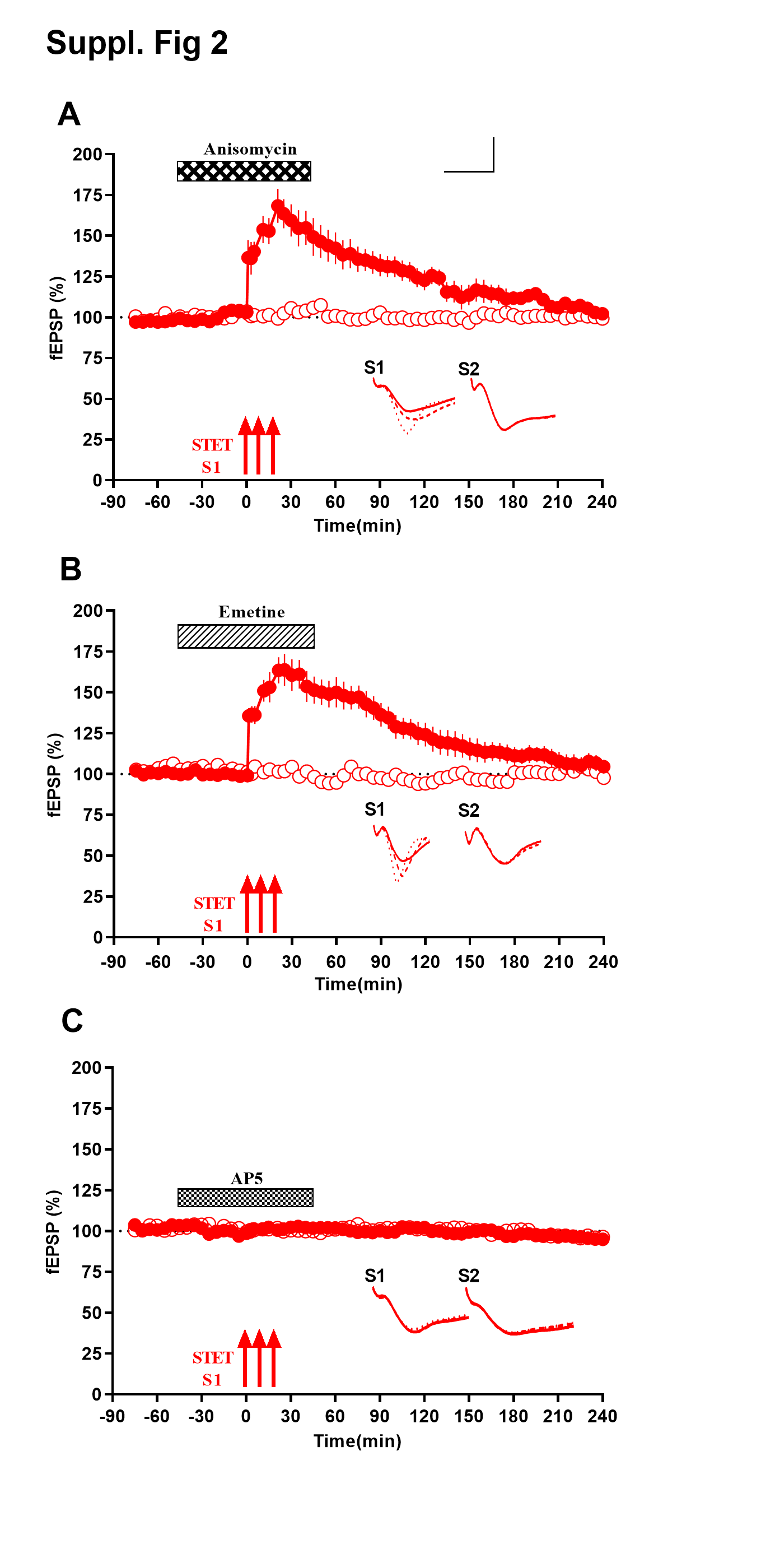

Supplement: Supplementary file 2 [file ACEL-19-e13046-s002.tif]

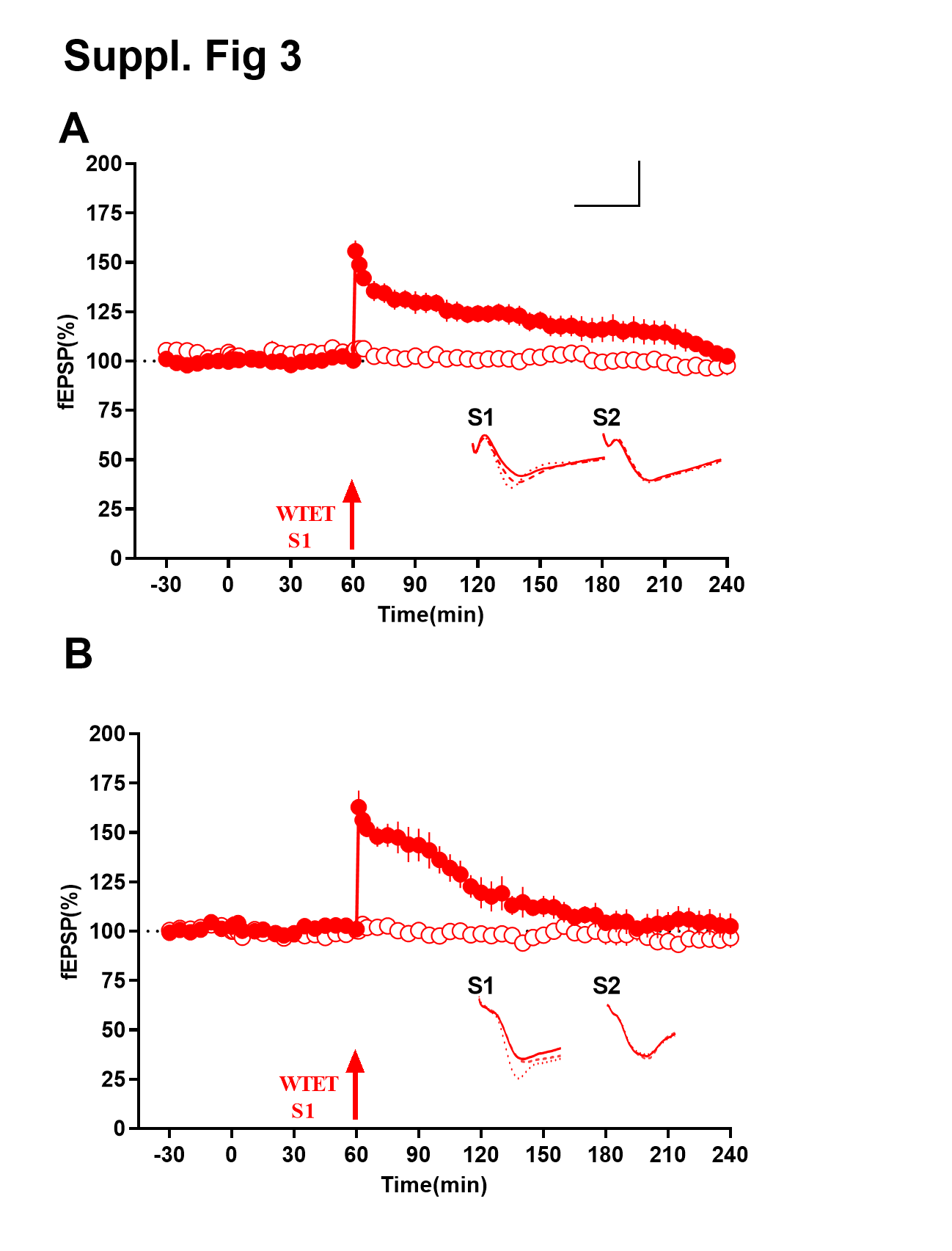

Supplement: Supplementary file 3 [file ACEL-19-e13046-s003.tif]

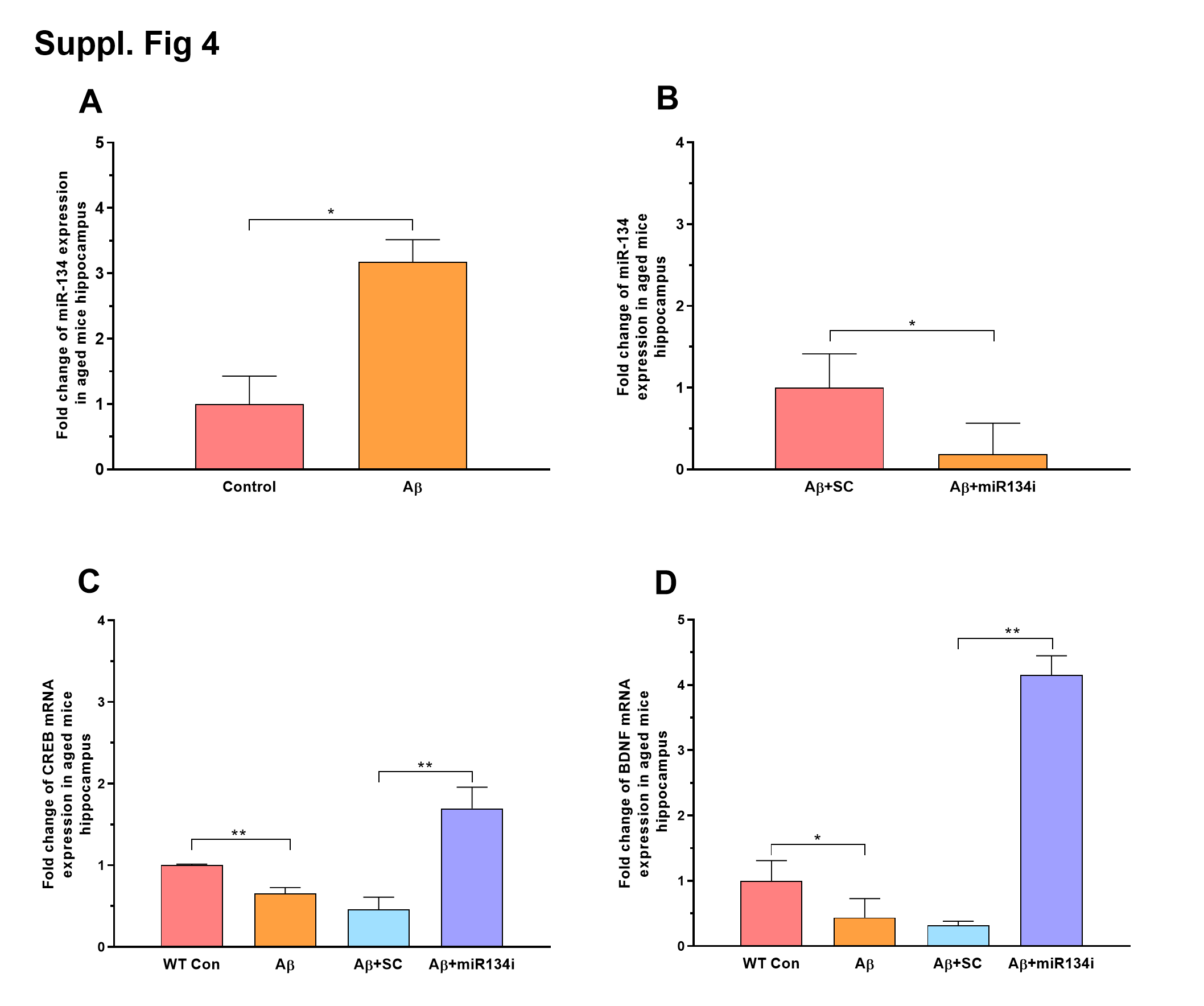

Supplement: Supplementary file 4 [file ACEL-19-e13046-s004.tif]

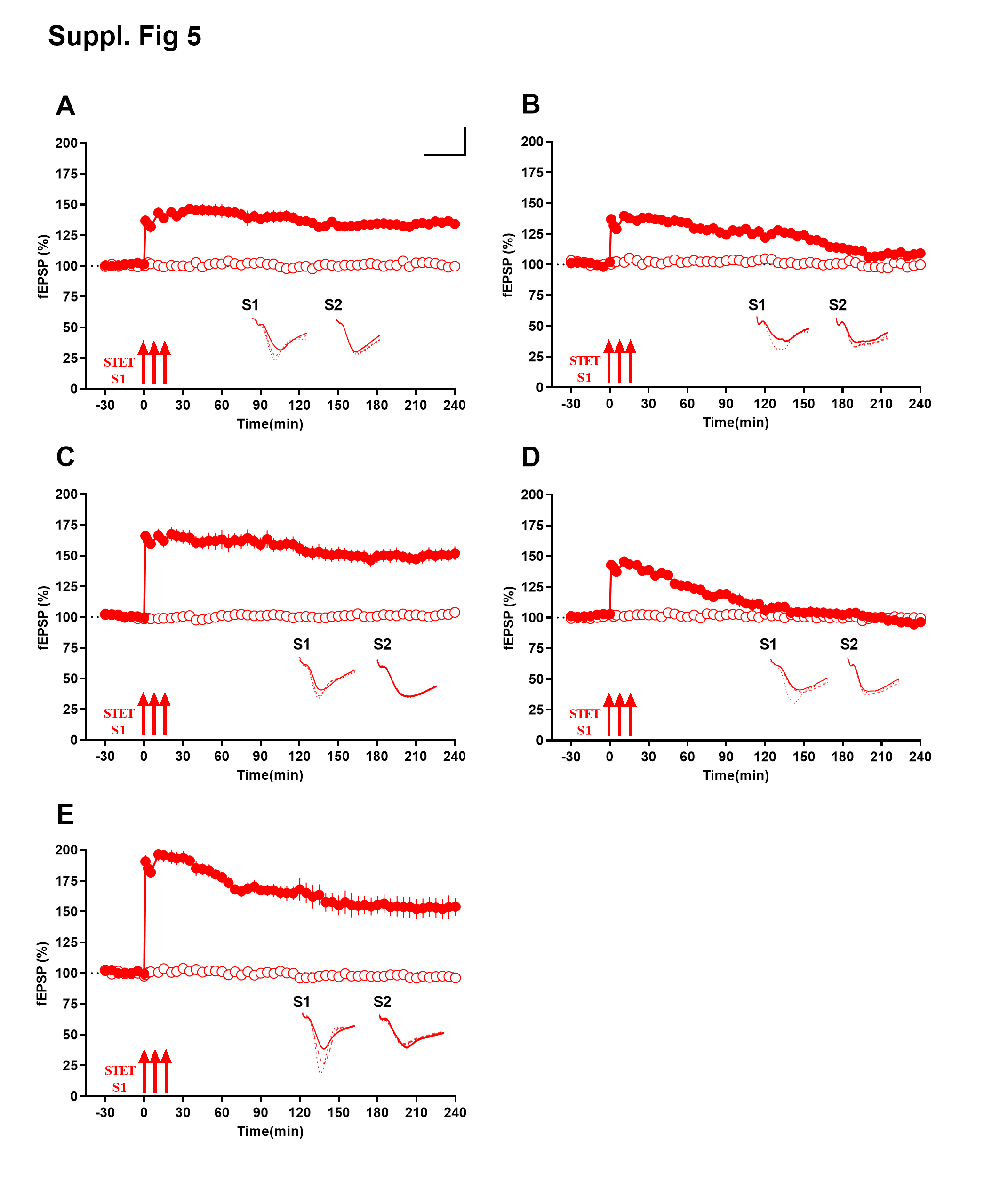

Supplement: Supplementary file 5 [file ACEL-19-e13046-s005.tif]

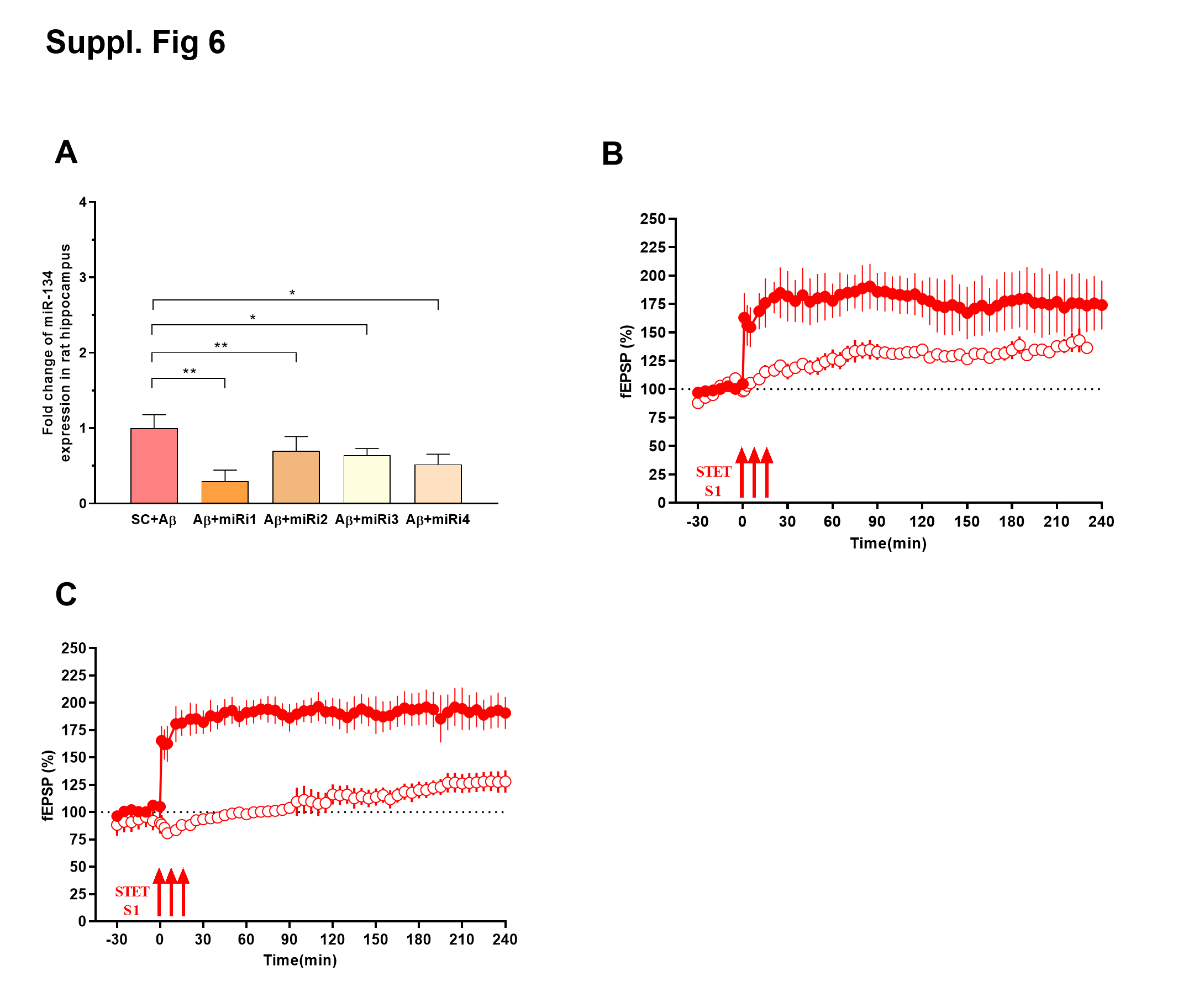

Supplement: Supplementary file 6 [file ACEL-19-e13046-s006.tif]
